# The effect of chemical erosion on mechanical properties and fracture of sandstone under shear loading: an experimental study

**DOI:** 10.1038/s41598-019-56196-2

**Published:** 2019-12-27

**Authors:** Cancan Chen, Shoujian Peng, Shankang Wu, Jiang Xu

**Affiliations:** 1 State Key Laboratory of Coal Mine Disaster Dynamics and Control, Chongqing University, Chongqing, 400044 China; 2 State and Local Joint Engineering Laboratory of Methane Drainage in Complex Coal Gas Seam, Chongqing University, Chongqing, 400044 China

**Keywords:** Engineering, Solid Earth sciences

## Abstract

In order to study the effect of water-rock interactions on shear strength characteristics, we performed shearing tests under varying hydrochemical environments. Moreover, a custom meso-shear test equipment for coal rock was used for the tests. Through 3D scanning of the shear fractures and scanning electron microscope imaging, we studied the effect of different pH chemical solutions on the shear strength and fracture characteristics of sandstones. We obtained three main results. With increasing solution acidity or alkalinity, water-hemical solution corrosivity increases. Moreover, the shear strength of sandstones reduces almost linearly and the fracture surfaces become smoother. The erosive effect is evidenced by the decrease in fracture surface fluctuations, roughness and the high-order microbulges, and scaling of the grain structure. A collection of characteristic parameters, including the maximum height *S*_*h*_, the root mean square deviation *S*_*q*_, the area ratio *S*_*A*_, and the slope root mean square *S*_*∆q*_, can be used to quantitatively describe the rough and irregular texture of the fracture surface.

## Introduction

During engineering activities such as the discharge of waste liquid in injection wells^1^, geological sequestration of CO_2_^2,3^, or large-scale hydrofracturing using acidic solutions^4^, the rock mass is not only loaded but also etched and corroded by the chemical solutions in the ambient environment. This chemical exposure changes the rock mass’s mineral components, structure, and mechanical properties, thus threatening stability^5,6^.

When the rock of the porous medium is in an aqueous environment, the absorption and transportation of internal moisture is closely linked to the capillary action. And capillary water absorption is one of the most significant physical properties of rock, which is the best property to evaluate the penetration of water into rock materials^7^. Studies have shown that rock with increased porosity and thus increased absorption is more sensitive and less durable^8,9^. For example, N. Sengun had found that capillary water absorption coefficient (CWA) values have a linear relationship with total porosity and CWA values have an inverse relationship with ultimate compression strength. In addition, the higher the capillary water absorption and porosity, the worse are the negative influence on physical and mechanical properties of rock^10^. The water inhaled will produce such effects as lubrication, softening and crystallization, resulting in the reduction of rock strength and elastic modulus.

Interactions between the chemical solution and rock mass include physical and chemical activities. Compared with the physical negative influence of lubrication, softening, and argillization, water-rock chemical reactions have a greater impact on the mechanical properties of the rock mass^11^. For instance, the loss of strength, Young’s modulus, cohesion and internal friction angle is more obvious on rock samples immersed in different chemical solutions than these just saturated with pure water^12–15^. However, Miao *et al*. found the poisson’s ratio of granite increased after treatment with water and acidic solutions^16^. This is mainly due to the microstructural damage caused by chemical reactions between rock and chemical solution, which resulted in the macroscopic loosening and fragility of rocks.

Furthermore, The damage to the rock macro-mechanical properties caused by water-rock chemical reactions is closely related to the chemical properties of the solution, such as pH value, ion concentration, and ion components^17^. Some research have shown that neutral water(PH = 7) has the least effect on the strength and elastic modulus reduction and this reduction of strength and elastic modulus increased with the increasing in the acidic or alkaline degree of the solution with the same ionic concentration^15,18^. Besides, Feucht *et al*. had found that solution of intermediate ionic concentration produced the least mechanical weakening and the mechanical weakening increased with the ionic concentration increasing or decreasing of solutions^19^. This phenomenon was also observed by Feng *et al*.^18^. In addition, there is different influence on reduction of rock strength after the rock soaked into chemical solutions that contained different ionic components. For example, among chemical solutions of NaCl, CaCl_2_, and NaHCO_3_ with the same ionic concentration and pH value, NaCl and NaHCO_3_ solutions cases had the largest reduction and smallest reduction on strength of the limestone specimens, respectively^20^. But for sandstone, NaHCO_3_ and CaCl_2_ solutions produced the largest loss and smallest loss of strength, respectively. These are mainly due to the chemical reaction between solutions and main mineral components of rock samples^21^.

It is clear that rock mass is geological material with many initial internal micro-cracks and surrounds by complex hydrological environment. The change of internal microstructure of rock mass can cause the change of macro-mechanical properties^22^. During the process of water-rock chemical corrosion, an erosion of mineral particles and crystals, a weakening of particle bonds strength, and an alteration of the mineral components will cause the degradation of the rock macro-mechanical properties^23^. Therefore, it is important to study the micro-mechanism of rock mass under chemical corrosion. Over the past few decades, several methods have been employed to study the micro-mechanism of water-rock chemical reaction, including computerized tomography(CT)^24^, nuclear magnetic resonance spectroscopy(NMR)^25,26^ and scanning electron microscope(SEM)^27^. CT value and CT image obtained from the CT testing technique provides an effective mean to analysis of rock damage evolution^28^. Feng *et al*. analyzed the damage evolution of sandstone by adopting CT images and CT values and defined a damage variable based on the chemical corrosive influence^21^. Through the NMR, pore volume changing with time can be measured after rock immersed into different chemical solutions. It can be found that number of small pores and large pores both increased significantly due to the dissolution of minerals and erosion of microstructure^15,29^. By mean of SEM, it can be found that surfaces of sandstone that subjected to water-rock reaction are more rough and show little gouge^19^. Besides, the bigger particles of granite broken into a number of smaller, fragmented particles and the microstructures became looser under the SEM^16^. However, the observation field of SEM is very limited, it is difficult to analyze the whole fracture morphology of rock sample.

Previous research have indeed made some achievements, however, the existing studies of water-rock chemical reactions so far mainly have been focused on macro mechanical properties and micromorphology of rock under uniaxial and triaxial compression tests. Few research have emphasized shear-resistant mechanical performance and macroscopic fracture morphology of rock after corroded by chemical solutions. Therefore, this paper utilizes a self-developed meso-shear test device for rock to conduct shear-fracture tests on sandstone corroded by chemical solutions with different pH values. Besides, a three-dimensional scanner is adopted to scan the shear and fracture surface, and quantify the surface smoothness and fracture characteristics through a software package. Furthermore, We analyzed the macroscopic impact of corrosive effects of the chemical solutions on the shear-resistant properties of sandstone using statistical methods and use a scanning electron microscope (SEM) to relate the macroscopic features to microstructural changes.

## Test Methods

### Sample preparation

**Figure 1:**
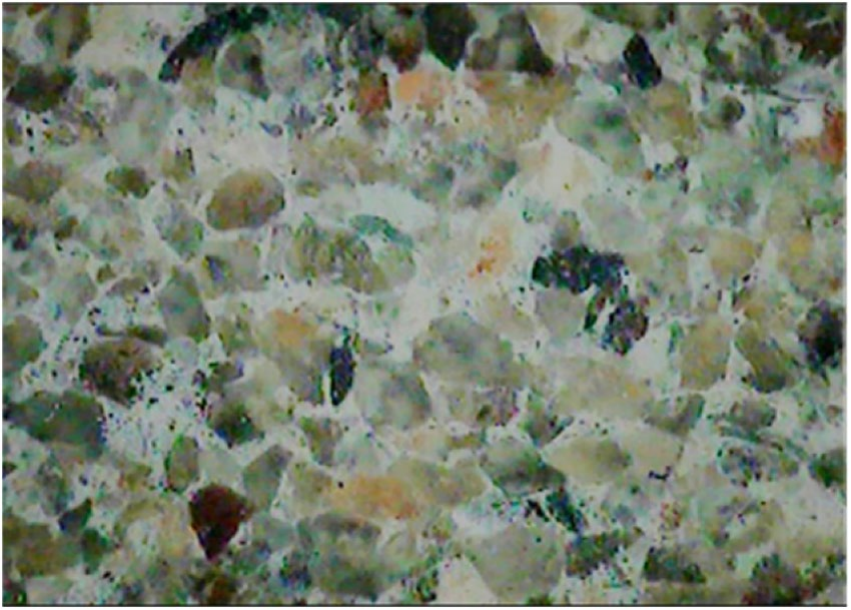
Microstructure images of sandstone samples.

**Figure 2:**
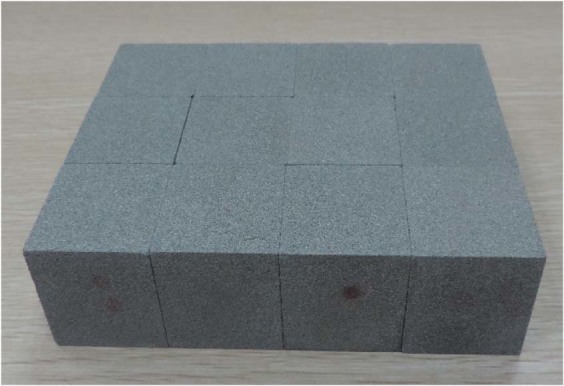
Experimental specimens.

The rock samples used in the test are upper Triassic Xujiahe (T_3_xj) sandstone, which is classified as terrigenius fine grain clastic sedimentary rock with a particle diameter of 0.1–0.5 mm. The main components of the sandstone include quartz, feldspar, flint, and muscovite, and an image of its microstructure is shown in Fig. 1. During the test, large-size complete sandstones were selected and incised to reduce the discreteness of the test specimen. The small-size sandstones were fabricated into cube specimens of dimensions 40 mm × 40 mm × 40 mm with wet processing. The processing precision was controlled within 0.02 mm. Figure 2 shows the fabricated specimens and Table 1 lists their basic physico-mechanical parameters.

**Table 1:** Physico-mechanical parameters of sandstone.

Density *ρ*/g·cm^−3^	Natural moisture content *ω*/%	Uniaxial compression strength *σ*_c_/MPa	Young modulus *E*/GPa	Poisson’s ratio *ν*	Cohesion *с*/MPa	Internal friction angle *φ*/°
2.28	4.67	55.97	11.89	0.37	12.82	61.42

### Preparation of water-chemical solution

In real environment, the pH value of underground water is generally 5.5–8.5. However, during waste liquid injection, the injected waste liquid may be strongly acid or alkaline; thus, we prepared a gradient of 5 pH values, namely, 2, 5, 7, 9, and 12. The preparation method for the water-chemical solution is as follows: First, we prepared a NaCl solution with pH = 7 and concentration of 0.1 mol/L. Second, we added 0.1 mol/L HCl and NaOH solutions to the NaCl solution to prepare NaCl solutions with pH values of 2, 5, 9, and 12^19^.

### Testing conditions

According to the test plan (see Table 2), three sandstone specimens were arranged under each type of testing condition so that a total of 21 samples were tested. After the specimen processing is completed, the samples were placed in ovens with a temperature of 105 °C for 24 h, cooled in a sealed environment, and then soaked in 150 ml of solutions with pH of 2, 5, 7, 9, and 12 and pure water for 14 days. During the soaking, we regularly monitored the change in solutions’ pH value (the measurement data for the solution with pH of 5 was missed) and kept the environment sealed during the whole process.

**Table 2:** Test scheme for shearing.

Chemical solutions	pH value	Ionic concentration/(mol/L)	Corrosion time/d	Normal stress/MPa	Shear rate/(mm/min)
NaCl	2, 5, 7, 9, 12	0.1	14	0	0.02
Pure water	7	/			
Dry	/	/	/		

**Figure 3:**
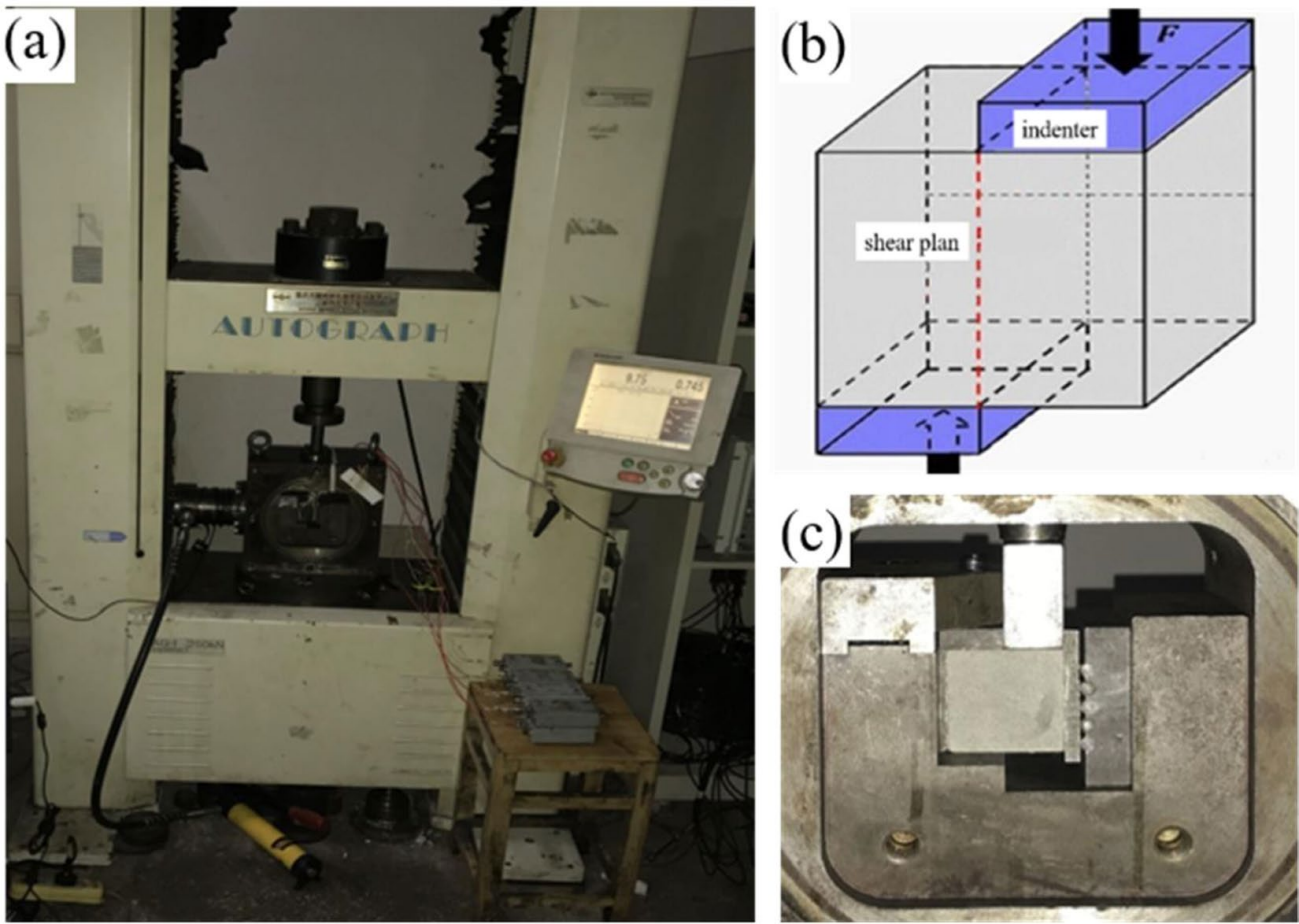
Loading system of rock shear testing device (**a**), shearing schematic (**b**), shearing fixture (**c**).

**Figure 4:**
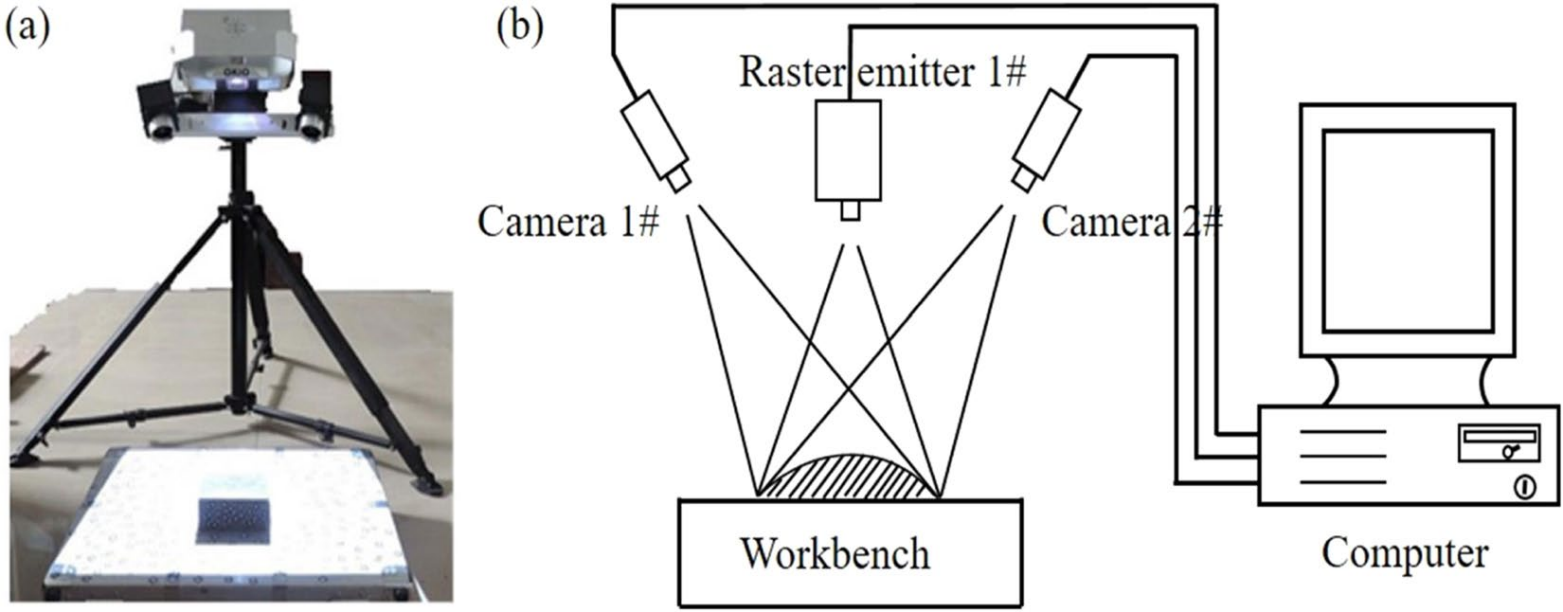
3D laser scanner device (**a**) Physical map (**b**) schematic diagram.

A custom meso-shear test device was adopted to conduct nonrestrictive shear tests^30^. Figure 3 shows the actual specimen installation and labels the forces exerted. After the test, a 3D laser scanner was employed to scan the three dimensions of the upper and lower shear-fracture surfaces (Fig. 4). Furthermore, We then used Matlab and other software to collect statistics and analyze the morphology characteristic parameters of the three-dimensional maps of the shear-fracture surface.

## Results and Discussion

### Change of pH value of the water-chemical solutions

**Figure 5:**
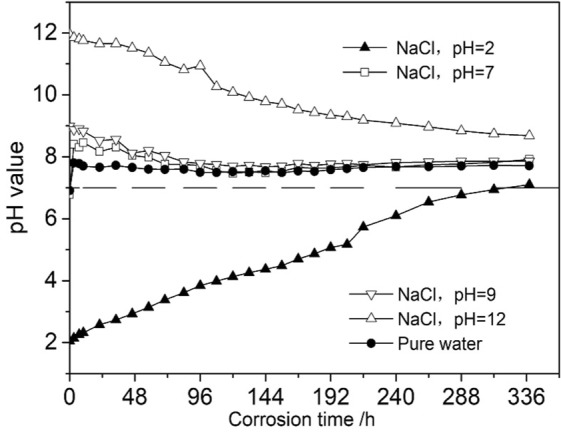
Change curves of pH values of water-hemical solution.

We used a PHS-2C acidimeter to measure the change of pH value of the chemical solutions during the whole soaking process. The measurements began when the specimen is completely soaked in the solution and the measurement frequency was by the change rate of the pH value. Figure 5 plots the change of pH values during the whole soaking process.

As Fig. 5 shows, the pH values of the solutions change rapidly at an early stage, which is mainly caused by the relatively large contact area between the chemical solution and specimen surface. Thus, the water–rock chemical coupling reaction is relatively strong. As time goes on, the etching and corrosive effects of the chemical solution on sandstone slowly extend from the surface to the interior and the pH value tends to change less, becoming stable after approximately 14 days. This means that the water–rock coupling reaction is strongly time-dependent; in other words, the chemical reaction will gradually weaken over the corrosion period and finally cease.

The pH value of all solutions finally approached 7 (neutral) as a result of the self-balancing feature of the solution’s pH value during the water-rock reaction. The pH value of pure water tends toward alkalinity, because the main component of the sandstone adopted in this test is aluminosilicate, which is weakly alkaline after hydrolysis.

### Effect of water-rock reaction on shear strength of sandstone

**Figure 6:**
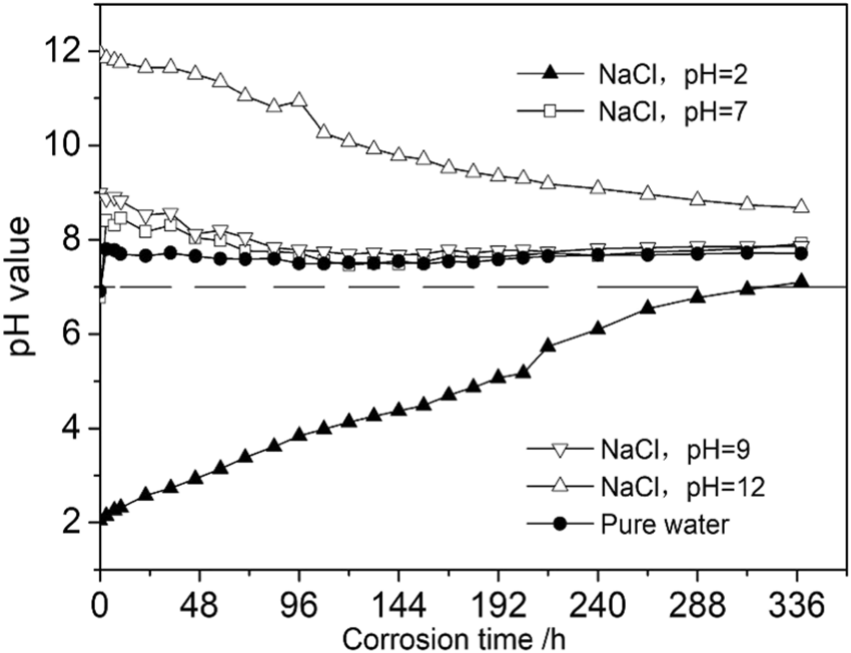
Shear-loading-displacement curves of sandstone under different etching solutions.

We conducted nonrestrictive shear tests for dry sandstone and sandstone etched in pure water and solutions with different pH values. Figure 6 shows the correlation curve between the shear stress and shear displacement obtained from the test.

As Fig. 6 shows, the shapes of the various curves are basically consistent. The samples experience shear failure at three stages: pore and fissure compaction, elastic deformation and nonstable fracture, and development. The shear strength of dry sandstone is 13.67 MPa, obviously higher than the samples that were soaked. The sandstone etched in NaCl solution with a pH of 12 had the lowest shear strength of 6.30 MPa, which demonstrates that the water–rock chemical coupling action greatly degraded the shear strength of the specimens.

Among all the etched sandstone samples, the sandstone etched by pure water had the highest shear strength of 9.25 MPa. The sandstone etched in NaCl solution with the same pH as pure water has a shear strength of 9.18 MPa, which is close to the above value. In addition, the two types of sandstones have similar shear-stress–shear-displacement curves, which means that the Na^+^ and Cl^−^ ions in 0.1 mol/L NaCl solution have less impact on the shear characteristics of sandstone.

**Figure 7:**
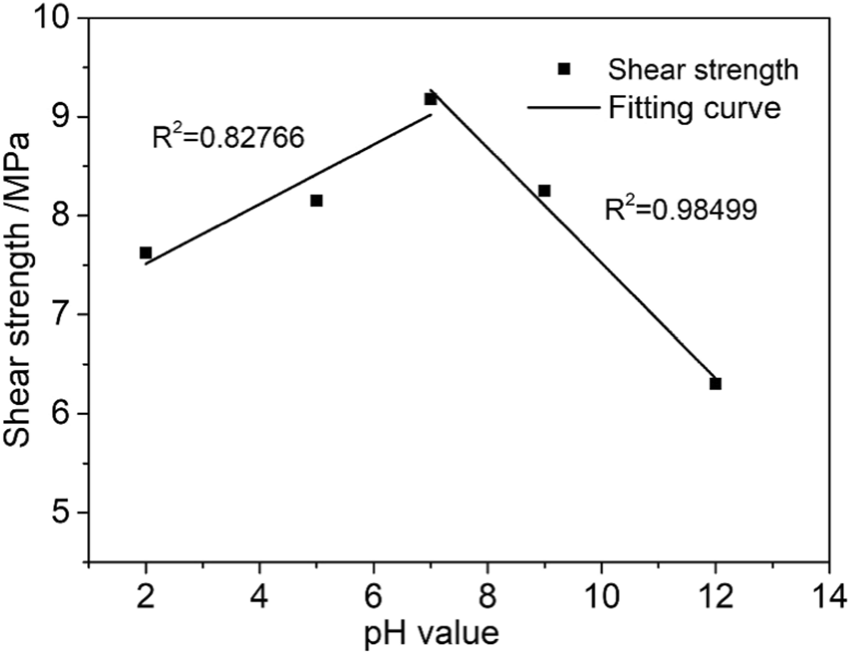
Shear strength-pH fitting curves.

Figure 7 shows a fitted curve for the relation between the shear strength of sandstone and pH value of water–chemical solution. As the figure shows, the shear strength of sandstone increases linearly as the acidity of etching solution weakens (pH = 2, pH = 5, pH = 7). Moreover, as the alkalinity of the etching solution increased (pH = 7, pH = 9, pH = 12), the shear strength of sandstone decreased linearly.

To describe the effect of the water-rock chemical coupling reaction on the shear strength of sandstone better, we classified the sandstone damage into two categories based on the chemical-damage model defined by Tang^31^. Type *A* damage *D*_A_ and Type *C* damage *D*_C_ are categorized according to the severity of the change of the rocks’ uniaxial compressive strength caused by water-chemical action:

1 $${D}_{A}=1-S/{S}_{0}$$

2 $${D}_{C}=1-S/{S}_{w}$$

where *S* indicates the shear strength of sandstone etched in NaCl solutions with different pH values, *S*_0_ indicates the shear strength of dry sandstone, and *S*_w_ indicates the shear strength of sandstone etched in pure water.

**Figure 8:**
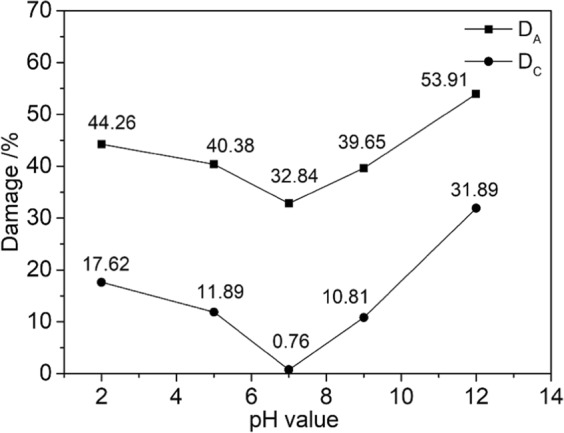
Damage-pH curves.

Type *A* damage includes physical and chemical damage to the rock structure, such as softening, argillization, and lubrication. Type *C* damage refers to the chemical damage to the rock caused by the change of the solution’s pH value. Figure 8 plots these two damage types versus the pH of the etching solution.

Figure 8 shows that as the acidity or alkalinity of the chemical solution increased, both types of damage gradually become serious. The alkaline solution degrades the rock more than the acid solution, which means that the sandstone is more sensitive to alkaline solutions. According to the plot of *D*_A_ against pH values, chemical solutions have a very obvious effect on the shear strength of sandstone. If the damage approaches 50% during engineering activities, the stability of the local rock mass may be compromised, causing geological disturbance and threatening the safety and people and property. According to the plot of *D*_C_ against pH values, the change of the solution’s pH value could cause significant chemical damage, which suggests that the pH value of the chemical solution is closely related to the strength of water–rock chemical coupling action and the corresponding weakening of the rock strength.

### The features of fracture surface and analysis of the SEM graph

**Figure 9:**
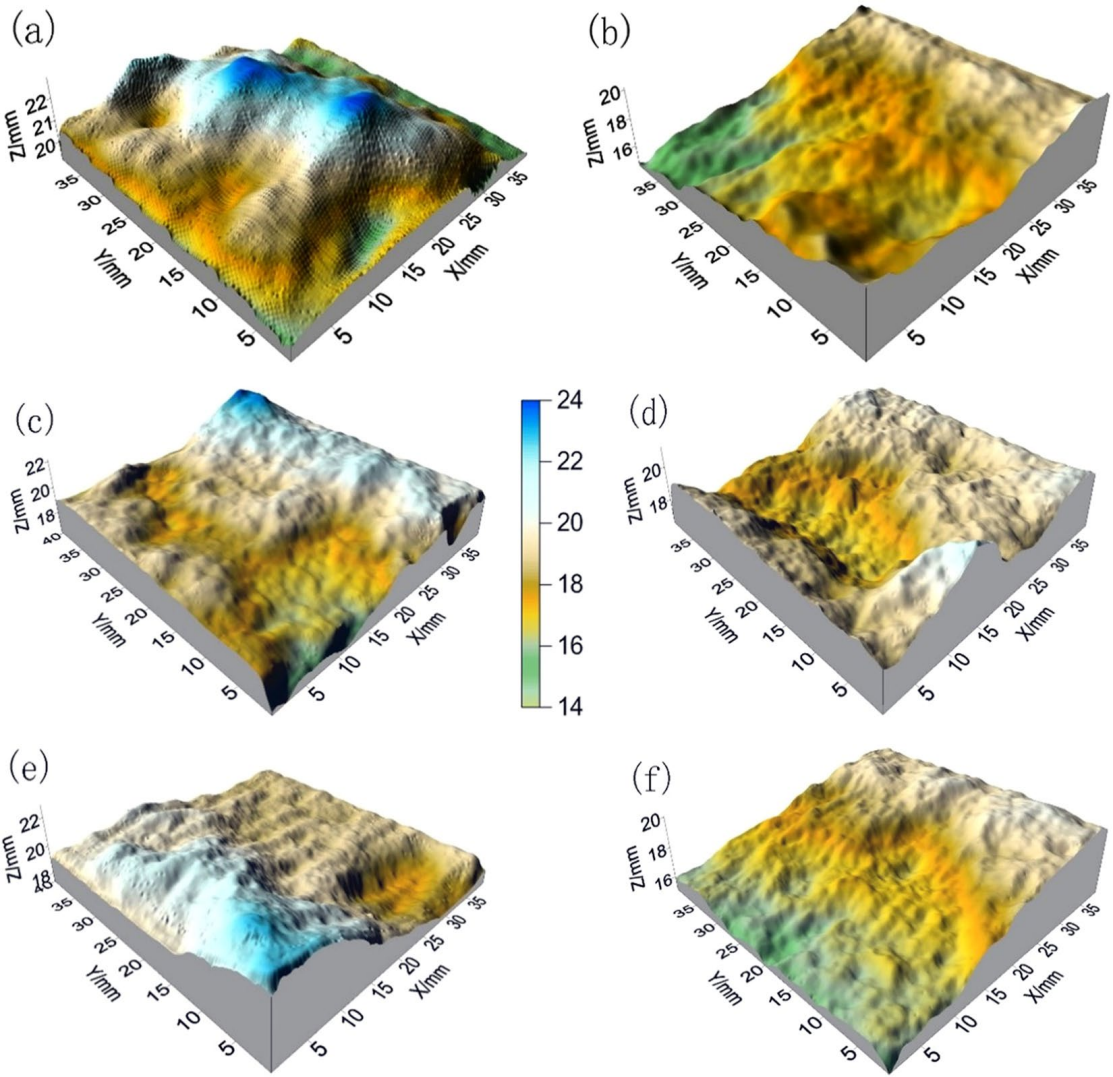
Three-dimensional graphs of sandstone fracture surface soaked in different etching solutions (**a**) dry, (**b**) pH = 2, (**c**) pH = 5, (**d**) pH = 7, (**e**) pH = 9, (**f**) pH = 12.

A three-dimensional scanning system (Fig. 4) was used to scan the stereometric and three-dimensional structure of the shear-fracture surface of the sandstone. The scanning results are shown in Fig. 9.

Figure 9 clearly shows that compared with the shear-fracture surface of sandstone etched in solutions, the shear-fracture surface of dry sandstone has a steeper topography (color gradient, see Fig. 9(a)) and more gullies, contained irregular uplifts in the middle area, and presented more obvious irregular microbulges on the surface. These features suggest that the shear-fracture surface fluctuates more and is more rough. The extension and perforation of the shear-fracture surface is more complex, the extension of internal fracture is more circuitous, and the shear resistance is larger during shear failure. For shearing fractures of sandstone under etching solutions, with increasing of acidity (pH = 7, pH = 5, pH = 2), the shearing fracture of sandstone has fewer steep portions and becomes smoother. The microconvexities on the surface also disappear gradually. The alkaline corrosion effect is similar to that of acidic corrosion. With increasing alkalinity of the etching solution, the shear-fracture surface of sandstone fluctuates less, the roughness decreases, and obvious water–chemical damage is evident.

**Figure 10:**
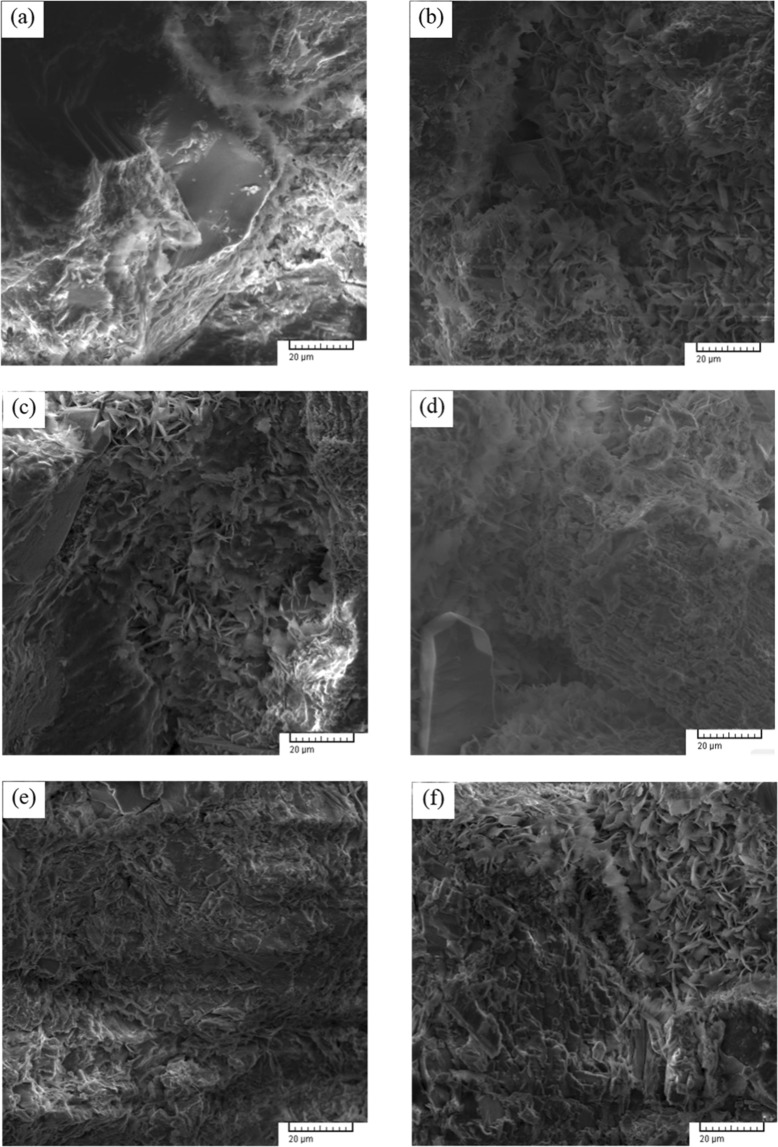
SEM photomicrographs of specimens with different corrosion damage (**a**) dry, (**b**) pH = 2, (**c**) pH = 5, (**d**) pH = 7, (**e**) pH = 9, (**f**) pH = 12.

In order to observe the microstructure of the mineral granules after the sandstone is corroded by the chemical solution, the samples under different test conditions were imaged by electron microscope, and an SEM graph of the samples is shown in Fig. 10.

As shown in Fig. 10, the mineral particles on the surface of dry sandstone connect compactly and flatly. However, for sandstone etching with a neutral solution, mineral grains were clearly separated in a loose cell-like pattern, and the clay mineral content between particles was seriously corroded. With the increase of the acidity or alkalinity of etching solution, the mineral particle morphology tends to be scale shaped. This morphology indicates that some particles dissolve by corrosion or argillization in the water; thus, the strength of the particles weakens and the cohesive forces between them are weakened.

### Analysis on morphological characteristics of shear-fracture surface

In order to describe the changes to the morphological characteristics of the sandstone shear-fracture surfaces, we quantified the corrosion effects of the chemical solution with a set of characteristic parameters. These characteristics can be divided into height and texture features of the surface morphology. The former may represent height fluctuations and the slope at each point on the fracture surface, and the latter may capture position between each point of the fracture surface and their correlation^32^. The combination of both can best describe morphology characteristics of fracture surface. Many of the characteristic parameters are inclusive. Based on the least-square plane, auxiliary software was applied to extract coordinate data for the shear fracture. Moreover, Matlab was used to select the height-characteristic parameters of the surface topography, the maximum profile height S_h_, profile mean square root deviation S_q_, texture characteristic parameters profile area ratio S_A_, and the slope mean square root S_∆q_.

The parameters are defined as follows:

1. The maximum profile height *S*_*h*_:
  3 $${S}_{h}=max(|{S}_{pi}|+|{S}_{mj}|)$$
  where *S*_pi_ (i = 1, 2,…) represents the distance from the highest peak to the base level for the *i*^*th*^ profile peak and *S*_*mj*_ (*j* = 1, 2,…) represents the distance from the lowest point to the base level in the *j*^*th*^ profile valley.
2. Profile mean square root deviation *S*_*q*_:
  4 $${S}_{q}=\sqrt{\frac{1}{m\times n}\mathop{\sum }\limits_{i=1}^{m}\mathop{\sum }\limits_{j=1}^{n}{Z}_{i,j}^{2}}$$
  where, *m* and *n* represent the number of points on the length and width of the sampling area and *Z*_*i*,*j*_ represents the distance from the profile point to the base level at coordinate (*i*, *j*) on the surface profile. When the profile point is above the base level, *Z*_*i,j*_ is positive.
3. Profile area ratio *S*_*A*_:
  5 $${S}_{A}={A}_{t}/A$$
  where *A*_*t*_ represents the extended area of the fracture surface and *A* represents the projected area of the fracture surface.
4. Slope mean square root *S*_*∆q*_:

6 $${S}_{\Delta q}=\sqrt{\frac{{\sum }_{i=1}^{m-1}{\sum }_{j=1}^{n-1}[{({Z}_{i+1,j}-{Z}_{i,j})}^{2}+{({Z}_{i,j+1}-{Z}_{i,j})}^{2}+{({Z}_{i+1,j+1}-{Z}_{i+1,j})}^{2}+{({Z}_{i+1,j+1}-{Z}_{i,j}+1)}^{2}]}{2(m-1)(n-1){\Delta }^{2}}}$$

where *Δ* represents the sampling spacing. The rest of the symbols are the same as above.

After soaking in water solution with different pH values, the three-dimensional morphology characteristic parameters of the shear-fracture surfaces are listed in Table 3.

**Table 3:** Three-dimensional parameters of fracture surface.

NO	*Z*_*max*_	*Z*_*min*_	*S*_*h*_	*S*_*q*_	*S*_*A*_	*S*_*∆q*_
pH = 2	2.5787	−3.1159	5.6946	1.1544	1.0308	0.1034
pH = 5	2.8105	−2.9978	5.8083	1.3092	1.0336	0.1099
pH = 7	4.0447	−3.4385	7.4832	1.4647	1.0667	0.1118
pH = 9	3.2823	−2.2125	5.4948	1.1256	1.0361	0.1107
pH = 12	1.9852	−1.7372	3.7224	0.7915	1.0314	0.1022
Dry	3.0907	−4.2185	7.3092	2.0638	1.0444	0.1222

**Figure 11:**
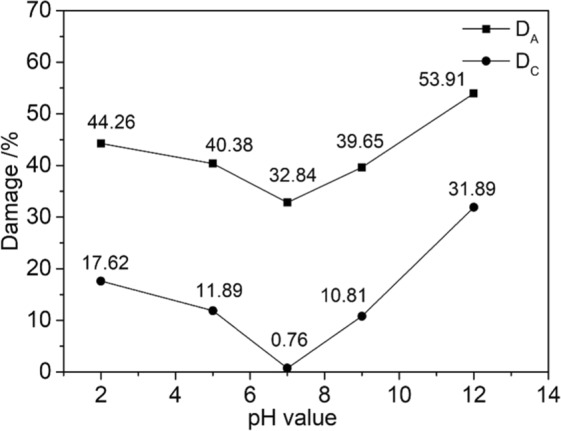
The correlation of three-dimensional height parameters and pH values.

**Figure 12:**
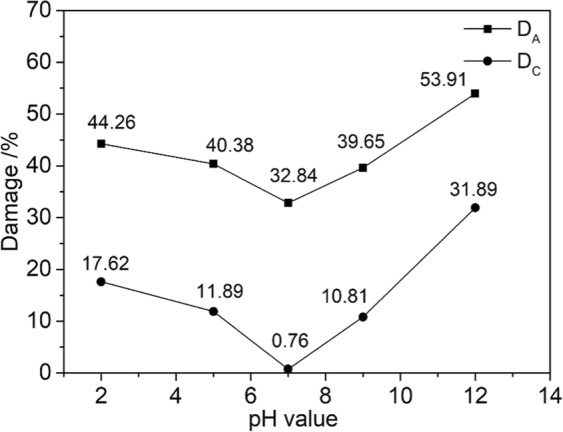
Correlation of three-dimensional texture parameters and pH.

Fig. 11 shows the correlation curve of the height parameters of the shear-fracture surface *S*_*h*_, *S*_*q*_, and the pH value of the chemical solution. Figure 12 shows the correlation curve of the texture parameters of the shear-fracture surface *S*_*A*_, *S*_*∆q*_, and the pH value of solution.

From Fig. 11 with pH = 7 as the demarcation point we observe that as the pH value of the solution increases or decreases, the maximum profile height *S*_*h*_ of the shear-fracture of sandstone decreases, indicating that the maximum fluctuation range of the fracture surface decreases gradually. The curve of the profile mean square root deviation *S*_*q*_ follows the same trend. The profile mean square root deviation fully describes the discreteness and volatility of the profile points on the fracture surface, which may decrease with increasing acidity or alkalinity solution, indicating that the discreteness and volatility of the profile points on the fracture surface decrease gradually. Analysis of the two height-characteristic parameters of the shear-fracture surface shows that higher acidity or alkalinity decreases the degree of surface fluctuation on the sandstone shear-fracture surface, implying that the fracture surface is more smooth. The plot also shows that alkaline corrosion smoothens the fracture surface more than acidic corrosion, which also confirms that sandstone is more sensitive to alkaline solutions. Figure 12 shows that as the acidity or alkalinity increases, the profile area ratio on the sandstone shear-fracture surface approaches 1 and the slope mean square root decreases, indicating that the folds on the fracture surface are fewer and tend to be more smooth.

In summary, with increasing acidity or alkalinity in the etching solution, the degree of fluctuation and roughness on the shear-fracture surface of sandstone decrease gradually, indicating that chemical solution causes increasing erosion damage to the sandstone. This is because solutions with stronger acidity or alkalinity may generate strong water–rock interactions, and the internal particles of sandstone are broken down by dissolution. When the shear-load effects run through the whole fracture surface, the fracture surface is more smooth and flat and high-order microconvexities on the surface are also reduced. This smoothing is caused, on the one hand, by weakening of the cohesive force between particles. On the other hand, because the microconvex bodies on the upper and lower fracture surfaces experienced more contact abrasion during loading due to the degradation of mechanical properties.

### Analysis on water-rock chemical coupling mechanism

The chemical effects of chemical solutions on rock mainly include ion exchange, dissolution, corrosion, hydration, hydrolysis, and oxidation reduction. The etching solution we tested is 0.1 mol/L of NaCl solution, with ionic composition of Na^+^, Cl^−^, H^+^, and OH^−^. Because the effects of Na^+^ and Cl^−^ under this ion concentration are not obvious on the degradation of sandstone, we only need to discuss the mechanism behind the influence of H^+^ and OH^−^ ions (the pH value, in other words) on the chemical action that corrodes sandstone. The main components of the sandstone selected for this experiment were quartz, feldspar, and kaolinite. Therefore, the water–rock chemical coupling mechanism is discussed in terms of the reactive mode of these three in solution.

In case of acid soaking, the pH value of the solution rises. This is mainly caused by feldspar (k-teldspar, albite, and thiorsanite) and kaolinite reacting with H^+^ in the solution to consume H^+^ in the solution. The chemical reaction equation is as follows:

7 $$KAlS{i}_{3}{O}_{8}({\rm{K}}-{\rm{teldspar}}\,)+4{H}_{2}O+4{H}^{+}\to {K}^{+}+A{l}^{3+}+3{H}_{4}Si{O}_{4}$$

8 $$NaAlS{i}_{3}{O}_{8}({\rm{Albite}})+4{H}_{2}O+4{H}^{+}\to N{a}^{+}+A{l}^{3+}+3{H}_{4}Si{O}_{4}$$

9 $$CaA{l}_{2}S{i}_{2}{O}_{8}({\rm{Thiorsanite}})+8{H}^{+}\to C{a}^{2+}+2A{l}^{3+}+2{H}_{4}Si{O}_{4}$$

10 $$A{l}_{2}S{i}_{2}{O}_{5}{(OH)}_{4}({\rm{Kaolnite}})+6{H}^{+}\to 2A{l}^{3+}+2{H}_{4}Si{O}_{4}+{H}_{2}O$$

In case of alkaline soaking, the pH value of the solution decreases, and quartz and kaolinite are the main components to participate in the corresponding reactions. The chemical reaction equation is as follows:

11 $$Si{O}_{2}({\rm{Quartz}})+2O{H}^{-}\to Si{O}_{3}^{2-}+{H}_{2}O$$

12 $$A{l}_{2}S{i}_{2}{O}_{5}{(OH)}_{4}+5{H}_{2}O+2O{H}^{-}\to 2Al{(OH)}_{4}^{-}+2{H}_{4}Si{O}_{4}$$

In case of neutral soaking, the pH value of the solution inclines weakly toward alkalinity. This is because hydrolysis of quartz and feldspar tends to weak alkalinity. Even if the kaolinite reaction consumes OH ^−^, its influence on the pH value is negligible compared to the previous two reactions. The specific chemical reaction equation is as follows:

13 $$KAlS{i}_{3}{O}_{8}+8{H}_{2}O\to {K}^{+}+Al{(OH)}_{4}^{-}+3{H}_{4}Si{O}_{4}$$

14 $$NaAlS{i}_{3}{O}_{8}+8{H}_{2}O\to N{a}^{+}+Al{(OH)}_{4}^{-}+3{H}_{4}Si{O}_{4}$$

15 $$CaA{l}_{2}S{i}_{2}{O}_{8}+8{H}_{2}O\to C{a}^{2+}+2Al{(OH)}_{4}^{-}+2{H}_{4}Si{O}_{4}$$

16 $$Si{O}_{2}+2{H}_{2}O\to {H}_{4}Si{O}_{4}$$

17 $$A{l}_{2}S{i}_{2}{O}_{5}{(OH)}_{4}+5{H}_{2}O+2O{H}^{-}\to 2Al{(OH)}_{4}^{-}+2{H}_{4}Si{O}_{4}$$

According to the water-rock chemical equation, the mineral composition of sandstone is eroded by the soaking process. Moreover, the granular structure is weakened and loosened, resulting in degradation of the mechanical properties of the sandstone samples. As the pH value increases, the degree of grain corrosion increases and the cohesive forces between particles are weakened, thus resulting in intensified wear and smoother fracture surfaces.

In conclusion, the change of sandstone microstructure is closely related to macroscopic changes to the shear mechanical parameters. The mineral composition of the sandstone samples and their microstructural changes correspond with the degradation to mechanical parameters caused by chemical solution soaking.

## Conclusion

The following present several major conclusions drawn from the results and discussion above:

1. With increase of the acidity or alkalinity in water-chemical solution, the shearing strength of sandstone is in linear decrease, indicating that the stronger acidity or alkalinity in water solution, the more serious water-chemical damage inside the sandstone after soaking, resulting in decrease of mechanical properties of sandstone.
2. Shear-fracture morphology from three-dimensional scanning and SEM imaging showed that stronger acidity or alkalinity caused greater damage to the sandstone. High or low pH smoothed the fracture surface and caused the internal structure to be scaled in shape. This evidence indicates that etching effects exacerbated the dissolution, corrosion, and argillization of particles inside the sandstone and clay, thus weakening the sandstone’s internal structure of sandstone.
3. The three-dimensional morphological characteristic parameters *S*_*h*_, *S*_*q*_, *S*_*A*_, and *S*_*∆q*_, which were obtained with self-compiled Matlab scripts, can describe the degree of fluctuation and roughness of the fracture surface of sandstone quantitatively. With increasing acidity or alkalinity in the soaking solution, these characteristic parameters decrease gradually, indicating that the shear-fracture fluctuation gradually slows down. The roughness also decreases gradually, reflecting the influence of the water-chemical damage on the internal structure of sandstone.
